# Association between bisphenol A exposure and adiposity measures in children

**DOI:** 10.1097/MD.0000000000041065

**Published:** 2024-12-27

**Authors:** Cui Liu, Ying Liu, Jing Ning, Chunyan Wu, Xiuxia Lu, Yong Guo, Peisi He, Chuhui Qiu, Jieling Wu

**Affiliations:** a Department of Children’s Health Care, Guangdong Women and Children Hospital, Guangzhou Medical University, Guangzhou, Guangdong, China; b Department of Public Health and Preventive Medicine, School of Medicine, Jinan University, Guangzhou, Guangdong, China.

**Keywords:** bisphenol A, BMI z-score, children, obesity, skinfold thickness, upper arm circumference, waist-to-height ratio

## Abstract

Bisphenol A (BPA) is a chemical that has adverse effects on human health and may cause childhood obesity. Nevertheless, the association between BPA exposure and adiposity measures in children remains controversial, especially in young children. A cross-sectional study was conducted on 208 randomly selected children 4 to 6 years old attending preschools in Guangzhou, China. BPA exposure was assessed through ultra-high performance liquid chromatography-tandem mass spectrometry of urinary samples. Childhood adiposity measures were determined, including body mass index, waist circumference, skinfold thickness, and upper arm circumference. BPA was detected in all urinary samples, and the median urinary BPA concentration was 0.54 (interquartile range, 0.05–5.81) μg/L. In the adjusted models, children with higher urinary BPA concentrations had a higher body mass index z-score (β = 0.471; 95% confidence interval [CI]: 0.303, 0.640), and they were at a greater risk of overweight or obesity (odds ratio [OR] = 3.308; 95% CI: 2.151, 5.089). Higher urinary BPA concentrations were associated with an elevated waist-to-height ratio (β = 0.007; 95% CI: 0.002, 0.012), and they were at a higher risk of abdominal obesity (OR = 1.711; 95% CI: 1.102, 2.655). Higher urinary BPA concentrations were also associated with increased upper arm circumference and skinfold thickness in the adjusted models (β = 0.546; 95% CI: 0.278, 0.813; β = 0.702; 95% CI: 0.139, 1.266, respectively). Higher urinary BPA concentrations in children 4 to 6 years old were associated with a greater risk of overweight/obesity and abdominal obesity. BPA exposure might increase the risk of obesity in children. Further investigations are needed to confirm this association and explore the underlying mechanisms.

## 1. Introduction

Childhood overweight and obesity have reached a global epidemic.^[1]^ According to the Chinese National Survey on Students’ Constitution and Health survey, the prevalence of overweight and obesity among Chinese children and adolescents was estimated to be 20.5% and will continue to increase.^[2]^ Overweight and obesity in children may persist into adulthood, and such children are at a greater risk of cardio-metabolic diseases.^[1]^ The most significant risk factors for obesity are associated with an imbalance between physical activity and energy intake, but accumulating evidence suggests that exposure to environmental endocrine-disrupting compounds (EDCs) may play an important role in the development of childhood obesity.^[3,4]^

As a well-known EDC, bisphenol A (BPA) possesses estrogenic, antiestrogenic, antiandrogenic, and antithyroid properties.^[5]^ It is typically found in beverages and food stored in epoxy resin containers and polycarbonate plastic.^[6]^ BPA exposure is widespread in the environment. It can be detected in many consumer goods, including dental sealants, children’s toys, food and liquid storage containers, microwave ovenware, thermal paper receipts, and protective inner lining in food cans.^[7]^ BPA can be absorbed through skin contact, inhalation, and oral intake of food contaminated with BPA.^[8]^ Measurable concentrations of BPA are detected in urinary samples of almost all subjects from various countries. Indeed, international biomonitoring studies have confirmed that more than 90% of children are exposed to BPA in China, the United States, Australia, and Europe.^[9–12]^ Concern exists that children might be at a greater risk of exposure to some EDCs or more vulnerable to their effects than adults. The potential health-related influences of exposure to BPA in the critical period of early childhood development have gradually attracted scholars’ attention.

Studies suggested that childhood exposure to BPA was associated with obesity. Indeed, the obesogenic potential of BPA has been previously proved.^[13]^ BPA can promote adipocyte differentiation, proliferation, and hypertrophy.^[14]^ Although the experimental studies are convincing, the available epidemiological evidence is inconsistent.^[15,16]^ In addition, the association between BPA and childhood obesity may vary with exposure time, sex, and pubertal status.^[17]^ Previous studies mainly focused on the association between urinary BPA concentration and childhood obesity at school age,^[18]^ including abdominal obesity. Still, there are few studies on whether urinary BPA concentration is associated with the risk of obesity in young children (4–6 years old). Early childhood is a stage of rapid physical development. The metabolic functions in this period are not perfectly developed and are more vulnerable to endocrine disruptors.

Therefore, the present study aimed to evaluate the association between urinary BPA concentrations and adiposity measurements in children 4 to 6 years old. The results could have implications for preventing overweight and obesity in children and the subsequent detrimental consequences in adulthood.

## 2. Methods

### 2.1. Study design and population

This cross-sectional study was conducted on 208 preschool children aged 4 to 6 years in Guangzhou, China, between September and November of 2020. We selected 1 preschool and used a stratified sampling method to select 3 classes from each age group randomly. The parents were informed of the study and consented to their child’s participation.

The inclusion criteria were 4, 5, or 6 years, being taller than 95 cm, and being heavier than 10 kg. The exclusion criteria were a diagnosis of major medical conditions (such as liver, kidney, or endocrine diseases), incomplete data, recent use of drugs, or receiving treatment for acute or chronic diseases.

### 2.2. BPA exposure assessment

In the present study, 10-mL polypropylene tubes were used to collect urinary samples from a single non-fasting point on-site. The sample was immediately stored at −20 °C. The urinary BPA concentration was detected by ultra-high performance liquid chromatography-tandem mass spectrometry based upon the improved method proposed by Yang et al.^[19]^ According to previous studies,^[20,21]^ 4 mL of urine was mixed with 0.50 mL of phosphorous acid buffer, and 40 µL of β-glucuronidase (Sigma Chemical Co., St. Louis, MO). The mix was aliquoted in glass tubes and incubated for hydrolyzation. The hydrolyzed samples were extracted twice with ethyl acetate:n-hexane (1:1) (HPLC grade; Dikma, New York, NY). After centrifugation at 3000 × *g*, the upper organic phase was transferred to glass tubes and evaporated with nitrogen gas. The residue was dissolved in a 40% acetonitrile–water solution for analysis, which was carried out at the Department of Clinical Mass Spectrometry Laboratory. The limit of detection for BPA was 0.2 μg/L. The inter-batch precision recovery of BPA in urine was 92.6% to 120.0%, and the intra-batch precision was 3.54% to 7.02%. The BPA concentration was standardized to creatinine measured using an automatic biochemical instrument (7100; Hitachi, Tokyo, Japan). According to the potential nonmonotonic dose–response effects of BPA,^[22]^ BPA concentrations in the 25th, 50th, and 75th percentiles were used as the dividing points.

### 2.3. Data collection

The caregiver was the child’s primary caregiver, that is, the 1 who spent the longest time at home with the child every day except sleeping. Physical measurements were taken by trained researchers who were unaware of each child’s BPA exposure level. Body mass index (BMI) was calculated as weight in kilograms divided by height in squared meters. BMI varies greatly depending on age and gender. The age- and sex-standardized BMI z-scores were used according to the growth reference standards of the World Health Organization (WHO) in 2007,^[23]^ in which children who were ≥ 85th percentile for age and sex were classified as overweight/obese. Waist circumference (WC) was obtained to the nearest 0.1 cm by placing a tape at a point midway between the lower borders of the rib cage and the iliac crest at the end of expiration, and the average of duplicate measures was taken.^[24]^ The waist-to-height ratio (WHtR) was calculated as WC in centimeters divided by height in centimeters. WHtR ≥ 0.5 indicated abdominal obesity.^[25]^ Triceps skinfold thickness was obtained to the nearest 0.1 mm using a skinfold caliper. Upper arm circumference was measured by placing a flexible tape midway between the olecranon and acromial process on the upper right arm. Besides, triceps skinfold thickness and upper arm circumference were analyzed as continuous variables due to the lack of reference thresholds for Chinese children.

This study attempted to adjust for several possible confounding factors, collected by questionnaires to each child caregiver. Demographic characteristics were included in the questionnaire, including the child’s sex and age, parents’ age and BMI, and caregiver’s educational level (classified as primary, secondary, or tertiary). Other characteristics, such as gestational week, mode of delivery, birth weight, and breastfeeding duration (categorized as < 6 vs ≥6 months), were also obtained. Physical activity was determined by inquiring the caregiver how many hours the child would spend on medium and high-intensity exercises in a day outdoors. The caregivers were requested to consider any form of activities that could cause tachypnea or sweating, such as, but not limited to, running, riding a bicycle, riding a kick scooter, skipping rope, kicking a shuttlecock, kicking a ball, or all sports and games that require effort such as crawling, walking, running, jumping, and playing ball.

Exposure to environmental tobacco smoke, as a risk factor for the development of childhood metabolic syndrome, was obtained by asking whether there were smokers (yes/no) among the child’s caregivers.^[26,27]^ Information on children’s dietary habits was indirectly reflected through the consumed junk foods and the frequency of eating takeout. Junk food consumption was attained by inquiring the caregiver how often (per week) the child ate fried food (such as chips and fried chicken), sweet foods (such as beverages, ice cream, and candy), processed meat products (such as canned meat and sausage), and fast foods, such as instant noodles, hamburger, and pizza. The frequency of eating takeout was determined by inquiring the caregiver how many times the child ate takeout at a restaurant in the past week.

### 2.4. Statistical analysis

SPSS 23.0 software (IBM Corp., Armonk, NY) was used for data analysis. Continuous and categorical variables were respectively described as mean ± standard deviation (SD) and percentage. Continuous data with a skewed distribution were log-transformed. Urinary BPA concentrations were divided into Q1 to Q4 from low to high. One-way analysis of variance (ANOVA) and the chi-square test were used to compare the differences in adiposity measures and risk for obesity in each group, respectively. The significance level was set at a *P*-value of <.05.

Multiple linear regression analysis was performed to assess the association of urinary BPA concentration with continuous outcomes, including BMI z-score, WHtR, triceps skinfold thickness, and upper arm circumference. A multivariable logistic regression analysis was used to examine the association of urinary BPA concentration with categorical outcomes (e.g., overweight/obese).

Confounding variables possibly associated with urinary BPA level and/or anthropometric results or that changed the coefficient for BPA exposure in the model by > 10% were included.^[18,28,29]^ Therefore, the final model was adjusted for the following confounding variables: urinary creatinine levels to account for body composition and urinary dilution^[18,30]^; sex, as a potential effect modification of BPA concentration (continuous)^[29]^; caregiver’s educational level (classified as primary, secondary, or university), representing the socioeconomic status; children’s diet, including duration of early breastfeeding, junk food consumption (categorized as < 3 vs ≥3 times a week), and the frequency of takeaway (categorized into no vs <3 vs ≥3 times a week), considering the effect of diet on urinary BPA concentration and obesity; physical activity, as a potent predictor of anthropometry in children, which could be used as a confounding factor unpredictably, given the possible influence of physical activity on the excretion and metabolism of EDCs.^[31]^ The time spent watching TV daily was considered to assess behavioral risk (the association with obesity was previously mentioned in NHANES).^[26]^ Childhood passive smoking, as a risk factor for the development of childhood metabolic syndrome, was also included in the model.^[27]^

## 3. Results

### 3.1. General characteristics

Of the 208 children (4–6 years old) enrolled in this study, 126 were male. The mean (SD) age and BMI were 5.2 (0.7) years and 15.4 (1.9) kg/m^2^, respectively. Moreover, 72.1% of children were born by vaginal deliveries, with an average birth weight of 3.2 (0.38) kg. Parents’ BMI was 23.4 (3.0) and 21.3 (2.6) kg/m^2^, respectively. About 40% and 29% of caregivers had completed primary and secondary studies, respectively; the remaining 23% had completed college education. The mean (SD) time spent on outdoor activities and television watching by the children were 1.2 (0.4) and 1.0 (0.5) hours, respectively. Most children (72.6%) were breastfed for > 6 months, while 27.4% were breastfed for < 6 months. About 25.5% of children had passive smoking, and 74.5% did not. Around half of children did not eat takeout, while 38% and 12% ate takeout < 3 times and ≥3 times a week, respectively. About 39.4% of children consumed junk foods < 3 times a week, while 60.6% of children consumed junk foods ≥ 3 times a week (Table 1).

**Table 1:** Sociodemographic characteristic.

Childhood characteristics	Mean ± SD, n (%)	Parental characteristics	Mean ± SD, n (%)
Child age	5.2 ± 0.7	Maternal age	32 ± 5
Sex		Maternal BMI	21.3 ± 2.6
Boy	126 (60.6)	Paternal age	34 ± 5
Girl	82 (39.4)	Paternal BMI	23.4 ± 3.0
Delivery modes		Caregiver’s educational level	
Eutocia	150 (72.1)	Primary	83 (39.9)
Cesarean	58 (27.9)	Secondary	68 (32.7)
Gestational week	39 ± 1	University	57 (27.4)
Birth weight	3.2 ± 0.38	Childhood anthropometric measures	Mean ± SD/ n (%)
Breastfeeding duration (months)		Child weight (kg)	19.1 ± 3.9
<6 months	57 (27.4)	Child height (cm)	110.8 ± 6.4
≥6 months	151 (72.6)	BMI (kg/m^2^)	15.4 ± 1.9
Childhood passive smoking		BMI z-score	-0.05 ± 1.18
Yes	53 (25.5)	BMI z-score categorized	
No	155 (74.5)	Underweight/normal weight (<85th percentile)	148 (71.2)
Time spent on physical activity (hours/day)	1.2 ± 0.4	Overweight/obese (≥85th percentile)	60 (28.8)
Childhood television watching (hours/day)	1.0 ± 0.5	Waist circumference (cm)	51.4 ± 4.6
Childhood urinary creatinine (mg/dL)	67.6 ± 39.9	Waist-to-height ratio (WHtR)	0.46 ± 0.03
Junk food consumption		WHtR categorized	
<3 times a week	82 (39.4)	Normal (WHtR < 0.50)	167 (80.3)
≥3 times a week	126 (60.6)	Abdominal obesity (WHtR ≥ 0.50)	41 (19.7)
The frequency of takeaway		Upper arm circumference (cm)	16.5 ± 1.8
Not eat	104 (50.0)	Triceps skinfold (mm)	9.5 ± 3.4
<3 times a week	79 (38.0)		
≥3 times a week	25 (12.0)		

The children’s mean (SD) BMI z-score and WHtR were −0.05 (1.18) and 0.46 (0.03), respectively. The prevalence of overweight/obesity (BMI z-score ≥ 85th percentile) and abdominal obesity (WHtR ≥ 0.5) was 28.8% and 19.7%, respectively (Table 1). The mean (SD) values of WC, upper arm circumference, and triceps skinfold were 51.4 (4.6) cm, 16.5 (1.8) cm, and 9.5 (3.4) mm, respectively.

### 3.2. Adiposity measures in different urinary BPA concentrations

**Figure 1.**
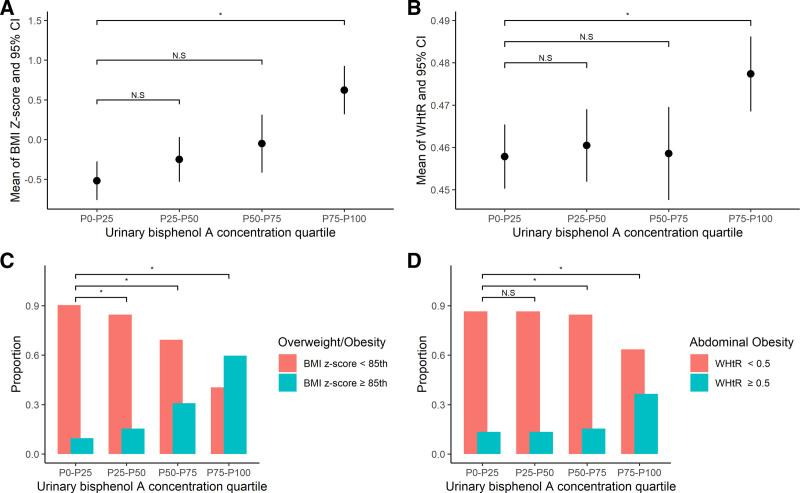
Urinary BPA exposure quartiles (Q1–Q4) and adiposity measures in children: (A) BMI z-score, (B) waist-to-height ratio, (C) proportion of obesity/overweight, and (D) proportion of abdominal obesity. *Compared with quartile 1, *P* < .05.

The median urinary BPA concentration was 0.54 μg/L (interquartile range, 0.05–5.81 μg/L). Children’s BPA exposure levels were divided into quartiles. BMI z-score, WHtR, overweight/obesity, and abdominal obesity significantly differed among the quartiles. Compared with Q1, the BMI z-score and WHtR were significantly higher in Q4. Compared with Q1, the children in Q2, Q3, and Q4 were at an increased risk of overweight/obesity, and those in Q3 and Q4 were at an increased risk of abdominal obesity (*P* < .05) (Table 2, Fig. 1).

**Table 2:** Urinary BPA exposure quartiles (Q1–Q4) and adiposity measures in children.

Urinary bisphenol A concentration quartile*	BMI z-score	Waist-to-height ratio (WHtR)	Overweight/obesity (BMI z-score ≥ 85th, n = 208)	Abdominal obesity (WHtR ≥ 0.5, n = 208)
	$\bar{x}\pm \;s$	$\bar{x}\pm \;s$	N (%)	N (%)
1	-0.519 ± 0.895	0.458 ± 0.028	5 (9.6)	7 (13.5)
2	-0.251 ± 1.035	0.461 ± 0.032	8 (15.4)†	7 (13.5)
3	-0.052 ± 1.339	0.459 ± 0.040	16 (30.8)†	8 (15.4)†
4	0.622 ± 1.117†	0.47± 0.033†	31 (59.6)†	19 (36.5)†
*F/χ* ^2^	11.278	3.993	38.040	12.485
*P*	.000	.009	.000	.006

* Quartile 1: values ≤ 0.29 μg/L; quartile 2: values of 0.30–0.54 μg/L; quartile 3: values of 0.55–1.03 μg/L; quartile 4: values ≥ 1.04 μg/L.

† Compared with quartile 1, *P* < .05.

### 3.3. Associations between urinary BPA concentration and adiposity measures

In the crude (unadjusted) models, children with higher urinary BPA concentrations had increased BMI z-score (β = 0.471; 95% confidence interval [CI]: 0.303, 0.640) and were at a greater risk of overweight or obesity (odds ratio [OR] = 3.308; 95% CI: 2.151, 5.089). After adjusting for confounding factors, such as urinary creatinine, sex, birth weight, parental BMI, caregiver’s educational level, breastfeeding duration, junk food consumption, the frequency of takeaway, physical activity, childhood television watching, and childhood passive smoking, the association remained almost unchanged (β = 0.436; 95% CI: 0.259, 0.612; OR = 3.675; 95% CI: 2.202, 6.133) (Table 3).

**Table 3:** The associations between urinary BPA concentration and adiposity measures in children.

BPA	BMI z-score		Waist-to-height ratio (WHtR)		Triceps skinfold thickness (mm)		Upper arm circumference (cm)		Overweight/obesity (BMI z-score ≥ 85th)		Abdominal obesity (WHtR ≥ 0.5)	
	β (95% CI)	*P*-value	β (95% CI)	*P*-value	β (95% CI)	*P*-value	β (95% CI)	*P*-value	OR (95% CI)	*P*-value	OR (95% CI)	*P*-value
Model 1	0.471 (0.303, 0.640)	.000	0.007 (0.002, 0.012)	.009	0.590 (0.070, 1.110)	.026	0.614 (0.357, 0.872)	.000	3.308 (2.151, 5.089)	.000	1.679 (1.128, 2.499)	.011
Model 2	0.478 (0.303, 0.653)	.000	0.008 (0.003, 0.013)	.003	0.641 (0.101, 1.181)	.020	0.622 (0.354, 0.890)	.000	3.460 (2.206, 5.429)	.000	1.743 (1.152, 2.636)	.008
Model 3	0.436 (0.260, 0.611)	.000	0.007 (0.002, 0.012)	.012	0.682 (0.132, 1.232)	.015	0.558 (0.289, 0.827)	.000	3.307 (2.086, 5.242)	.000	1.678 (1.105, 2.548)	.015
Model 4	0.436 (0.263, 0.609)	.000	0.007 (0.002, 0.012)	.009	0.695 (0.144, 1.246)	.014	0.559 (0.294, 0.824)	.000	3.538 (2.180, 5.741)	.000	1.718 (1.123, 2.629)	.013
Model 5	0.436 (0.261, 0.611)	.000	0.007 (0.002, 0.013)	.009	0.702 (0.144, 1.260)	.014	0.557 (0.289, 0.824)	.000	3.525 (2.157, 5.761)	.000	1.733 (1.128, 2.661)	.012
Model 6	0.436 (0.259, 0.612)	.000	0.007 (0.002, 0.012)	.011	0.702 (0.139, 1.266)	.015	0.546 (0.278, 0.813)	.000	3.675 (2.202, 6.133)	.000	1.711 (1.102, 2.655)	.017

For all models, n = 208. Continuous variables and categorized variables were presented as β (95% CIs) and OR (95% CIs), respectively.

Model 1: naturally log-transformed urinary BPA concentrations. Model 2: adjusted for urinary creatinine. Model 3: further adjusted for sex and birth weight. Model 4: further adjusted for caregiver’s educational level (classified as primary, secondary or university) and parental BMI. Model 5: further adjusted for breastfeeding duration (<6 months vs ≥6 months), junk food consumption (<3 times a week vs ≥3 times a week), and the frequency of takeaway (not eat/ <3 times a week/ ≥3 times a week). Model 6: further adjusted for physical activity (hours), childhood television watching (hours), and childhood passive smoking (yes vs no).

In the unadjusted models, higher urinary BPA concentrations were associated with increased WHtR values (β = 0.007; 95% CI: 0.002, 0.012) and a higher risk of abdominal obesity (OR = 1.679; 95% CI: 1.128, 2.499). The association remained almost unchanged after adjusting for all covariables (OR = 1.711; 95% CI: 1.102, 2.655). Similarly, higher urinary BPA concentrations were associated with elevated upper arm circumference and skinfold thickness in the fully adjusted model (β = 0.546; 95% CI: 0.278, 0.813; β = 0.702; 95% CI: 0.139, 1.266, respectively) (Table 3).

## 4. Discussion

The association between BPA exposure and childhood obesity has recently attracted clinicians’ attention, but inconsistent results were reported. The present study showed that higher urinary BPA concentrations in children 4 to 6 years old were associated with increased BMI z-score, WC, skinfold thickness, and upper arm circumference. Compared with the lowest quartile of the urinary BPA concentration group, the 2nd, 3rd, and 4th quartiles group were at an increased risk of overweight/obesity, and the 3rd and 4rth quartiles groups were at an increased risk of abdominal obesity. Therefore, higher urinary BPA concentrations in children were associated with a greater risk of overweight/obesity and abdominal obesity after adjusting for confounding factors. Consequently, early life exposure to BPA may be associated with the development of childhood obesity.

Similar to previous studies, all urinary samples in the present study had detectable levels of BPA.^[32]^ The present study indicated that the geometric average level of urinary BPA in children of 4 to 6 years old was 0.55 (range, 0.05–5.81) μg/L, which was higher than in Chinese school-age children (0.45 ng/mL),^[33]^ while it was lower than in American school-age children (3.60 μg/L for 6–11 years, ranging from 0.4–149 μg/L) and Turkish school-age children (5.0 ng/mL for 2–11 years, ranging from 1–24.4 ng/mL).^[9,34]^ Therefore, urinary BPA concentrations may vary among regions, and urinary BPA concentrations in Asian countries might be lower than in Western countries, consistent with a study conducted in South Korea.^[35]^ According to the National Biomonitoring data of the Republic of Korea,^[36]^ Koreans had lower urinary BPA concentrations than Canadians and Americans.

The present study also showed that the prevalence of overweight or obesity (28.8%) and abdominal obesity (19.7%) in children 4 to 6 years old in Baiyun District of Guangzhou was lower than in some developed countries.^[37,38]^ The prevalence of childhood overweight or obesity was 39%, according to the 2011 to 2012 Spanish National Health Survey.^[37]^ Another study found that 25% of Spanish children had abdominal obesity.^[38]^ In the present study, the prevalence of overweight or obesity was higher than the 25% reported in Changsha in 2017,^[39]^ while it was significantly higher than the 22.6% reported in 9 regions of China in 2012.^[40]^ Therefore, overweight and obesity are common in children, but certain regional differences exist. Still, the development trend of childhood obesity is not optimistic.

The present study revealed that higher urinary BPA concentrations in children of 4 to 6 years old were associated with increased BMI z-score, WC, skinfold thickness, and mid-upper arm circumference, and such children were at a greater risk of overweight/obesity and abdominal obesity. It suggests that BPA may have unfavorable effects on the development and growth of children. It is, therefore, essential to avoid the contact and absorption of BPA in daily life. When selecting items for children, it is suggested to avoid feeding bottles and foods containing BPA. Still, few studies have focused on the association between obesity and urinary BPA concentrations in children 4 to 6 years old. Notably, no study previously assessed the association between urinary BPA and mid-upper arm circumference, which has recently been considered an appropriate alternative for obesity screening in preschool children.^[41]^ The results of the present study are consistent with the findings of children of other ages in some previous epidemiologic studies. A recent study found that urinary BPA concentrations in obese children 2 to 11 years old were higher than in nonobese children from the same age group.^[34]^ Children of 4 years old had higher BPA concentrations and greater values of BMI z-score, WC, and skinfold thickness.^[42]^ In a Mexican cohort, higher BPA concentrations in girls 8 to 14 years old were associated with increased skinfold thicknesses.^[17]^ Another study demonstrated that children 6 to 18 years old with higher BPA concentrations had higher indices of generalized and abdominal obesity.^[43]^ On the other hand, some investigators reported inconsistent conclusions. They suggested that no association was to be found between BPA exposure in prenatal and early childhood stages and increased BMI in children of 2 to 5 years old,^[44]^ while some authors demonstrated that low BPA concentrations might lead to weight gain.^[45]^ BPA may produce a “U-shaped” effect.^[46]^ In addition, the dose and timing of BPA exposure may affect conclusions.^[28]^ Although previous epidemiological findings were inconsistent, the obesogenic potential of BPA has been proven. Studies demonstrated that BPA could promote adipogenesis through various mechanisms.^[47,48]^ The interaction between BPA and specialized nuclear receptors (e.g., steroid or thyroid hormone receptors) may be the main mechanism to promote adipogenesis.^[49]^ In future studies, it is essential to expand the sample size, including subjects from different regions, ages, and occupations, to confirm the relationship between BPA and the risk of being overweight or obese and abdominal obesity.

Childhood obesity reached epidemic proportions,^[1]^ and children with obesity are at a higher risk of cardio-metabolic diseases in adulthood.^[1]^ Hence, identifying any actionable factor that could be changed to decrease the prevalence of childhood obesity will have important public health benefits. BPA is found in specific products like epoxy resin containers and polycarbonate plastic, but they are considered nearly ubiquitous because of contamination.^[7]^ Nevertheless, restricting BPA exposure as much as possible should be conducive to helping prevent childhood obesity to some extent, as suggested by the present study.

The present study had some strengths. Firstly, a comprehensive assessment of childhood obesity-related indicators was conducted. Secondly, children of 4 to 6 years old were enrolled; such children were rarely well-studied, but they were more sensitive to the adverse influences of endocrine disruptors because of system immaturity. Finally, several relevant covariables were included in the model to reduce the effects of confounding factors.

### 4.1. Study limitations

Still, the limitations of the study should be pointed out. Firstly, the associations found in this study could not be considered causality as it was a cross-sectional study, which might result in bias in estimating the impact. Obese children may consume more foods containing BPA or have high levels of inside fat in which BPA has been detected.^[50]^ Secondly, urine samples were collected in our study to assess the exposure to BPA, which might be affected by various factors, including the time of sample collection and differences in diet and lifestyle of the study subjects. However, studies have shown that urinary samples collected on-site could also reliably reflect the BPA exposure level of the study population.^[51]^ Thirdly, although several potential confounding factors were involved in the model, the possibility of residual confounding caused by other unknown factors could not be ruled out. Fourthly, there were only 48 and 12 children with obesity and overweight, respectively, preventing subgroup analyses. Finally, the study was only conducted in 1 region, and its generalizability to the general population might be limited. Thus, it is necessary to conduct large-scale multi-center studies in the future.

In conclusion, higher urinary BPA concentrations in preschool children were associated with increased values of BMI z-score, WC, skinfold thickness, and mid-upper arm circumference, and such children were found to be at a greater risk of overweight or obesity and abdominal obesity. However, as it was a cross-sectional study, the associations found in the study cannot be considered as causality. Hence, it is essential to conduct further large-scale longitudinal research.

## Acknowledgments

The authors would like to acknowledge those participants who contributed to this research.

## Author contributions

**Conceptualization:** Jing Ning, Jieling Wu.

**Data curation:** Cui Liu, Ying Liu, Chunyan Wu, Chuhui Qiu.

**Formal analysis:** Cui Liu, Ying Liu, Chunyan Wu, Xiuxia Lu, Yong Guo, Peisi He, Jieling Wu.

**Methodology:** Jing Ning, Xiuxia Lu, Yong Guo, Chuhui Qiu.

**Validation:** Peisi He.

**Writing – original draft:** Cui Liu.

**Writing – review & editing:** Cui Liu, Ying Liu, Jing Ning, Chunyan Wu, Xiuxia Lu, Yong Guo, Peisi He, Chuhui Qiu, Jieling Wu.
